# Perennial Forages as Second Generation Bioenergy Crops

**DOI:** 10.3390/ijms9050768

**Published:** 2008-05-20

**Authors:** Matt A. Sanderson, Paul R. Adler

**Affiliations:** USDA-ARS, Pasture Systems and Watershed Management Research Unit, Building 3702, Curtin Road, University Park, PA 16802-3702; E-Mails: matt.sanderson@ars.usda.gov (M.A.S.); paul.adler@ars.usda.gov (P.R.A.)

**Keywords:** bioenergy crops, carbon sequestration, cellulosic ethanol, greenhouse gases, switchgrass

## Abstract

The lignocellulose in forage crops represents a second generation of biomass feedstock for conversion into energy-related end products. Some of the most extensively studied species for cellulosic feedstock production include forages such as switchgrass (*Panicum virgatum* L.), reed canarygrass (*Phalaris arundinacea* L.), and alfalfa (*Medicago sativa* L.). An advantage of using forages as bioenergy crops is that farmers are familiar with their management and already have the capacity to grow, harvest, store, and transport them. Forage crops offer additional flexibility in management because they can be used for biomass or forage and the land can be returned to other uses or put into crop rotation. Estimates indicate about 22.3 million ha of cropland, idle cropland, and cropland pasture will be needed for biomass production in 2030. Converting these lands to large scale cellulosic energy farming could push the traditional forage-livestock industry to ever more marginal lands. Furthermore, encouraging bioenergy production from marginal lands could directly compete with forage-livestock production.

## 1. Introduction

Second generation bioenergy crops [1], based on perennial forage crop species, are considered to be the future of the bioenergy industry and are the focus of intense research [2–4]. Compared with the first generation biofuels based on annual grain crops, perennial biomass crops require fewer inputs, produce more energy, and reduce greenhouse gas (GHG) emissions more than annual cropping systems [5]. Switchgrass (*Panicum virgatum* L.) is particularly compelling in North America because of its relatively low production inputs and costs, perennial growth habit, and adaptability to a broad range of growing conditions [6]. Perennial forage crops such as alfalfa (*Medicago sativa* L.), reed canarygrass (*Phalaris arundinacea* L.), napiergrass (*Pennisetum purpureum* Schumach.), and bermudagrass (*Cynodon* spp.) among many others, could also serve as perennial bioenergy crops for specific agroecoregions of North America [7].

These second generation bioenergy crops historically have been used for grazing and forage and were the original energy feedstocks for draft animal power [8]. Perennial forage crops currently supply the energy that fuels approximately 100 million ruminant animals on USA farms with a total estimated economic value of $US 39 billion [9]. The lignocellulose in perennial forage crops represents a vast and renewable source of biomass feedstock for conversion into the second generation of biobased products [10]. Indeed, the concepts of “fuel farming” are not new [e.g., 11–14]; however with new technologies and processes for biomass production and conversion nearing commercial reality [15], perennial forage crops could once again fuel agriculture.

Increasingly, farmers must consider managing for multifunctionality and include effects on environmental quality in their management decision-making [16]. Thus, grassland producers will need to consider managing for emerging ecosystem services such as enhancement of carbon (C) sequestration, mitigation of GHG emissions, and to capitalize on new opportunities, such as bioenergy production, to diversify the forage-livestock system to achieve these outcomes in the future [17]. In this paper we discuss some of the forage crops proposed for bioenergy use along with their environmental impacts, energy balance, research needs, and explore the implications of their widespread use.

## 2. Perennial Forage Species for Bioenergy

Of the many species of perennial forage crops available [18] only a few have been researched intensively for biomass. We briefly describe some of those species.

### 2.1 Switchgrass

In the USA, research on perennial bioenergy crops during the last two decades has focused on switchgrass, a C_4_ native warm-season perennial grass [4, 19]. Attributes of switchgrass desirable for bioenergy cropping include its demonstrated high productivity across many environments, suitability for marginal and erosive land, relatively low water and nutrient requirements, and positive environmental benefits [6, 20]. As potential disadvantages, switchgrass can be slow to establish, and productive stands often take two years to develop. ‘Alamo’ is a well adapted cultivar for the southern USA, and ‘Cave-in-Rock’ is a broadly adapted cultivar suitable for the mid-Atlantic, Northeast, and Midwest regions of the USA (Tables 1 and 2) [19, 20]. Newer varieties of switchgrass with improved biomass yield and chemical composition have been released [22–24]. Compared with other crop species, switchgrass has received relatively little plant breeding attention and most cultivars of this species are not far removed from native germplasm [20]. In some cases, cultivars of switchgrass could not be distinguished genetically from natural populations [25]. Thus, there is tremendous genetic variability and great potential for germplasm improvement [25–27].

### 2.2 Miscanthus

*Miscanthus*, a C_4_ grass native to Asia, is viewed as a model herbaceous biomass feedstock for Europe [28]. Although not used as a forage crop because of its morphology, *Miscanthus* is used as an ornamental plant in the USA. Researched extensively from northern to southern Europe (Table 1), the primary use of *Miscanthus* biomass is envisioned principally as a feedstock in combustion steam generating electrical plants [28]. *Miscanthus* has good cold tolerance for a C_4_ species and is winter hardy in temperate regions of Europe [29]. *Miscanthus* has a low requirement for N fertilizer because it efficiently recycles N between aboveground biomass and storage structures (rhizomes) belowground [30]. In most of Europe, *Miscanthus* grows from April until November; however, harvest of the previous year's biomass in February or March is recommended because moisture content and alkali elements in the standing biomass are reduced [31]. A drawback to *Miscanthus* use is that it must be established and propagated vegetatively via rhizome cuttings, which delays full production until the second or third year and also requires irrigation and energy inputs during greenhouse propagation [32]. A virtual comparison of *Miscanthus* production in Europe with switchgrass production in North America speculated that *Miscanthus* could produce twice as much biomass as switchgrass [33]. *Miscanthus* yielded 33% more biomass (18.1 vs. 14.1 Mg ha^−1^) than ‘Kanlow’ switchgrass grown on a heavy clay soil in southwestern Germany [34]. The authors cautioned, however, that Kanlow switchgrass may not have been well adapted to the soils and environment of the experimental site in Germany.

### 2.3 Reed Canarygrass

Scandanavian research on bioenergy crops has identified reed canarygrass as a highly productive perennial grass for northern Europe [28, 31]. Reed canarygrass is a C_3_ grass commonly used for hay and grazing that is well adapted to temperate agroecoregions and does well on wet soils [35]. Yields of reed canarygrass in Indiana, USA averaged 10 Mg ha^−1^ under low or high N fertilizer management (Table 1) [36]. Field trials in Iowa USA demonstrated biomass yields of 8.6 Mg ha^−1^ with 140 kg N ha^−1^ at two locations over 5 yr (Table 1) [37]. Some evidence exists for an efficient internal N recycling mechanism from shoots to roots in reed canarygrass [38]. Similar to switchgrass, reed canarygrass can be slow to establish, and yields are low in the seeding year. Reed canarygrass, however, can be an invasive species in native wetlands [39].

### 2.4 Alfalfa

Alfalfa is one of the world's oldest forage crops and is perhaps the highest value forage in North America [40]. Researchers in the 1990s envisioned alfalfa as a dual-use crop to simultaneously supply both biomass feedstock and a high quality animal feed [41]. In the dual-use system, leaves are separated for high-value, high-protein feed (an additional income stream) and the fibrous, lignified stems are combusted in an integrated gasification combined-cycle system to produce electricity. The proposed system recommended a two-cut harvest management to optimize economics, yield of stem and leaf, and wildlife habitat. Genetic selection efforts concentrated on lines developed for stiff stems with increased internode length to be grown under infrequent harvest (Table 1) [42]. Experimental biomass-type alfalfa germplasm has greater stem cell wall polysaccharide concentrations along with greater stem lignin concentrations, which contributed to greater stem dry matter yield and theoretical ethanol yields compared with hay-type alfalfas [43].

### 2.5 Other Species

Several other subtropical and tropical grasses have been evaluated as biomass crops in the southern region of the USA. The long, warm growing season and high rainfall in the southeastern region provide conditions for high yields ranging from 10 to 40 Mg ha^−1^ dry matter [44]. Bermudagrass (*Cynodon* spp.) is grown as a hay and grazing crop on about 4 million ha of the southern USA. Bermudagrass biomass yields have ranged from 13 to 20 Mg ha^−1^ at several locations in Georgia (Table 1) [45]. Napiergrass is a tall growing, perennial tropical grass with yields of up to 30 Mg ha^−1^ in Florida [46]. Eastern gamagrass (*Tripsacum dactyloides* L.) and prairie cordgrass (*Spartina pectinata* Link.) have also been explored as potential perennial grass feedstocks [47, 48].

There are many crops that could potentially be used to supply biomass feedstock to the emerging cellulosic (second generation) energy industry. This industry will require multiple sources of biomass and multiple biomass feedstocks for specific agroecoregions. The crops detailed here will probably be the first among many feedstocks to come from perennial forages.

## 3. Management for Bioenergy Cropping

Farmers are familiar with the agronomic management of perennial forage crops. Further, some of the machinery, technology, and infrastructure needed to plant, harvest, store, and transport forage crops can be used in bioenergy production. Details regarding specific management practices for bioenergy as they relate to forage production are discussed elsewhere [6, 7]. Principal management factors that influence biomass productivity and feedstock quality of a particular species include (i) rapid seedling establishment to reduce the time to productive stands, (ii) optimizing fertilizer inputs, and (iii) harvest management to optimize yield, persistence, and feedstock quality.

Establishment is a critical phase in forage and bioenergy crop production. Establishment failures substantially reduced switchgrass biomass and net energy yields in on-farm research [49]. Difficulties in stand establishment are often related to poor seed quality, improper planting depth, poor seedbed preparation, lack of weed control, and variable soil and weather conditions.

Fertilizer inputs must be optimized in biomass cropping systems because significant fossil fuel energy is used in the synthesis of the fertilizer. On-farm research with switchgrass production in the central Great Plains of the USA documented that fertilizer N accounted for 67% of the fossil fuel energy inputs to the biomass production system [49]. Inputs of N fertilizer also can be used as an indicator of potential environmental consequences of bioenergy cropping, such as leakage of nutrients to ground and surface waters along with atmospheric emissions. Nitrogen fertilizer recommendations for warm-season perennial grasses, such as switchgrass suggest a range of 10 to 12 kg ha^−1^ of N is required for each Mg of biomass harvested [50]. Crop rotations that include legumes to fix atmospheric N or mixtures of grasses and legumes potentially could reduce fertilizer N inputs into bioenergy cropping systems and improve their energy balance.

Warm-season perennial grasses internally recycle N from the aboveground shoots to the crown and roots in the fall for use in over-wintering and regrowth the following spring [51]. This mechanism enables an efficient use and reuse of N by the plant. *Miscanthus* had lower N fertilizer demand than reed canarygrass because of an efficient internal N recycling mechanism [30]. We lack critical information on when N recycling occurs within the plant, how much N recycles among plant organs, and quantitative data on how much recycling contributes to the N economy of a biomass energy crop.

Harvest management for biomass feedstock emphasizes yield and persistence. Producers managing stands that are dedicated to production of biomass feedstock may want harvest flexibility to respond to potential fluctuations in future feedstock markets [52].

Time of harvest affects switchgrass yield and varies with agroecological region. In the south-central USA, a single harvest in mid-September maximized biomass yields [21]. In the Central Plains of the USA, harvesting switchgrass at maturity (mid- to late August) maximized biomass yield. Delaying harvest until after a killing frost in October, however, reduced yields by 10 to 20% [53]. In the north-central USA a mid-August harvest reduced stand density compared with a fall harvest [54]. In the northeastern USA, mid- to late-summer annual yields were similar to spring-harvest yields after the initial high yield the first year of summer harvest [55].

Time of harvest also affects feedstock quality. In direct combustion systems, minerals in biomass can corrode and foul boilers [56]. The ash concentration of switchgrass decreases as it matures [57] leading to improved utility for conversion and potentially lower N requirements with a fall vs. summer harvest [53]. Delaying biomass harvest from fall to spring reduces mineral and water concentration of perennial forage grasses, but may also reduce yields [55].

## 4. Set-Aside, Marginal, and Abandoned Grasslands as Biomass Feedstock Resources

In addition to using new plantings of perennial bioenergy crops, other grassland-based resources such as set-aside lands along with marginal or abandoned lands could supply bioenergy feedstock. For example, cool-season grassland pasture on marginal lands in southern Iowa USA produced 0.8 to 8.2 Mg biomass ha^−1^ depending on site characteristics and environment (Table 1) [58].

Land in the Conservation Reserve Program (CRP; a government land set-aside program) has been suggested as a potential resource for biomass feedstock in the USA [59, 60]. The goal of the CRP is to remove land from crop production and establish long-term vegetation cover to prevent soil erosion, improve water quality, and enhance wildlife habitat. Of the 13.8 million ha of CRP land in the USA, about 6.8 million ha are potentially available for biomass feedstock production [59]. In the Northeastern USA, a two-year study demonstrated a standing feedstock supply of 6.6 Mg ha^−1^ on CRP and other conservation lands (Table 1) [61].

Maintaining the environmental benefits of the CRP is a concern when considering its potential for bioenergy production. This would include maintaining a perennial vegetative cover to prevent soil erosion and judiciously using fertilizers to obtain economic yields and not compromise water quality. Management practices suggested by research on CRP grasslands in the northern Great Plains of the USA included harvesting after a killing frost and adding fertilizer N at 56 kg ha^−1^ to maintain biomass production and stand persistence [62]. Biomass yields ranged from 2 to 8 Mg ha^−1^ at two locations during 3 yr. Carbon sequestration to a 90-cm depth on these same lands ranged from 2.4 Mg C ha^−1^ yr^−1^ with inorganic N fertilizer to 4.0 Mg C ha^−1^ yr^−1^ with manure application [63]. Mapemba *et al*. [64] applied economic models to estimate the cost of delivering a flow of feedstock from CRP lands in the southern Great Plains of the USA. Estimated feedstock costs ranged from US$29 to US$64 Mg^−1^ depending on conversion plant size, frequency of harvests, and the number of days suitable for harvest. Other management considerations for the use of CRP lands for biofuels in the future would include harvest management consistent with maintaining wildlife habitat.

### 4.1 Low-Input High-Diversity Prairie Systems

An intriguing development in using perennial crops for biomass is the so-called low-input high-diversity (LIHD) prairie approach [65]. Based on previous research into the productivity-biodiversity-ecosystem function relationship of ecosystems, estimates were made that low-input prairies could provide more usable energy and greater environmental benefits than corn-grain (*Zea mays* L.) ethanol or soybean (*Glycine max* L. Merr) biodiesel. Total CO_2_ sequestration (soil plus roots) rate under the high-diversity plots in Cedar Creek, Minnesota was 4.4 Mg CO_2_ ha^−1^ yr^−1^.

The LIHD approach relies on extremely low or no inputs of fertilizers and other materials and instead exploits increased productivity of multispecies plant communities on degraded lands. Criticisms of the research pointed out that the research was done under highly artificial conditions, (very small plots, hand weeding, and extremely high labor inputs) and did not represent realistic conditions [66]. These criticisms were rebutted [67]. Others, however, question whether degraded lands have the ability to produce the abundant quantities of biomass needed [68].

On-farm research on the northern Great Plains of the USA in large fields (3 to 9 ha) demonstrated that management of switchgrass as a bioenergy crop along with modest inputs of N fertilizer produced 93% more net energy than LIHD plots in Minnesota [49]. Switchgrass biomass yields in on-farm fields ranged from 5 to 11 Mg ha^−1^, whereas LIHD plots averaged 3.6 Mg ha^−1^ (Table 1). Soil C sequestration under switchgrass managed for bioenergy on the Northern Plains averaged 4.42 Mg C ha^−1^ yr^−1^ [69]. Switchgrass in monoculture performed poorly on the degraded soils of the Cedar Creek site; however, switchgrass in monoculture and binary mixture with a legume has been highly productive (5 to 16 Mg biomass ha^−1^ over 13 yr) on extremely degraded soils of abandoned strip mines in Virginia [70].

### 4.2 Integrated Bioenergy Crop-Production Systems

A perennial crop permanently dedicated to biomass feedstock production would seem to be an ideal goal because (1) there would be no annual re-establishment costs, (2) tillage would be eliminated, which would reduce inputs, costs, and soil erosion, and (3) a permanent vegetative cover would sustain soil conservation and water-quality protection. Perennials, however, are rarely permanent and some annual cropping or innovative combinations of annual and perennial bioenergy crops strategically deployed across the farm landscape and combined into synergistic rotations may be necessary in the future [71].

Combining annual bioenergy crops such as corn and sorghum (*Sorghum bicolor* L. Moench.) into rotations with perennial bioenergy crops, perhaps to jump-start the establishment phase, may benefit bioenergy cropping systems [71]. Including new cover crops, such as kura clover (*Trifolium ambiguum* L.), with annual crop systems as “living mulches” [72] could reduce environmental impacts. Innovative combinations of cool-season and warm-season annual crops could be the basis for dedicated biomass double cropping. Double cropping cool-season cereals with warm-season annuals, such as corn and sorghum was successful in the Midwestern USA if the cereal crop was removed as forage early in the spring to allow early planting of corn and sorghum for biomass [73].

**Figure 1.**
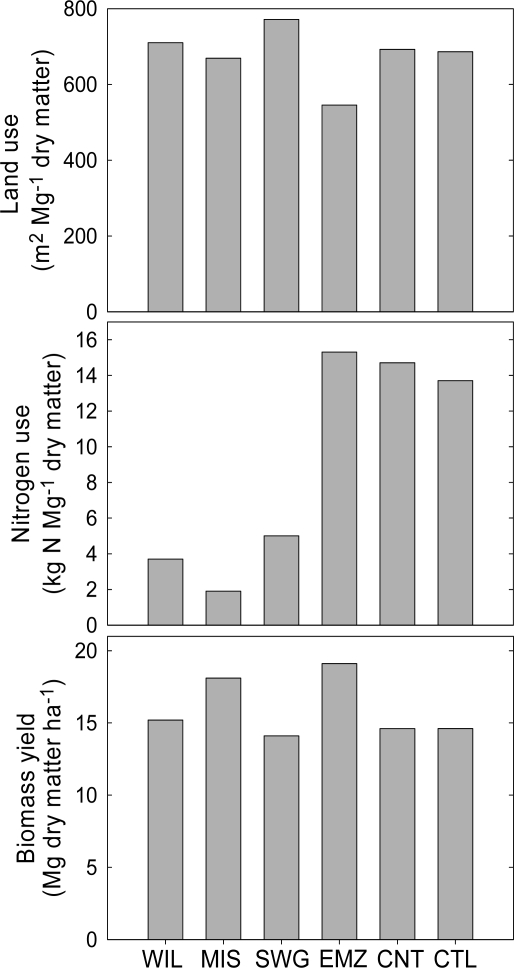
Biomass yield, nitrogen use efficiency, and land use efficiency for six cropping systems compared during 4 yr in southern Germany [34]. WIL, willow trees+27 kg N ha^−1^; MIS, *Miscanthus*+40 kg N ha^−1^; SWG, switchgrass+80 kg N ha^−1^; EMZ, energy maize +120 kg N ha^−1^ and grown with a grass cover crop; CNT, winter wheat, winter triticale, and oilseed rape grown in rotation with no-till methods; CTL, same crop rotation as CNT but grown with conventional tillage methods.

Results from cropping-systems research in southern Germany indicated that perennial biomass systems based on *Miscanthus*, switchgrass, or willows (*Salix schernii* E. Wolf x *viminalis*) could be as productive as energy maize with lower energy inputs (Figure 1) [34]. Nitrogen fertilizer was the most energy-intensive input and accounted for 41 to 64% of energy inputs for annual crops and 17 to 45% of inputs for perennials. Energy maize (grown with a grass cover crop for erosion and nutrient control) had the greatest land use efficiency because of high yields (19.1 Mg ha^−1^ of grain+stover), whereas the perennial crops had the greatest N use efficiency (Figure 1). Crop rotations based on annual cool-season plants [wheat (*Triticum aestivum* L.), triticale (Triticosecale x Wittmack), and oilseed rape (*Brassica napus* L.)] had the lowest energy use efficiency. Reducing cultivation intensity via no-tillage management in the cool-season crop rotations had a small effect on cropping system energy savings.

Relying on a diversity of crops and cropping systems in farm landscapes and larger scales (watersheds) would endow future bioenergy production systems with greater stability, resistance, and resilience to climatic and other environmental shocks [74, 75].

## 5. Economics and Environment

Currently, bioenergy produced from second generation cellulosic feedstocks costs more than fossil fuels [1]. Biomass yield, harvest and transport costs, conversion efficiency, and cost of fossil fuel used to produce the feedstock determine the economics of bioenergy production and vary among regions of the USA. Projected costs of switchgrass biomass range from $40 to $61 Mg^−1^ in Tennessee and $53 to $74 Mg^−1^ in Oklahoma [76]. Target costs estimated for economical production of ethanol from biomass feedstocks are about $38 Mg^−1^ [60]. The value of environmental benefits of bioenergy crops, however, may offset the price differential between biofuels and fossil fuels [10]. Results from watershed modeling studies suggest that payments of $11 to $27 Mg^−1^ of switchgrass biomass could be justified on the basis of projected water quality benefits from reduced sedimentation, erosion, and nutrient losses associated with growing a perennial grass [77].

The environmental benefits of bioenergy crops include increased soil quality, reduced losses of soil nutrients, soil C sequestration, protecting riparian zones [78], and recycling nutrients from sewage sludge, livestock manure, and bioconversion byproducts [71, 79–81] among others.

In the Chesapeake Bay region of the USA, concern over the potential for large-scale conversion of land to corn production for biofuels, with the potential attendant increase in soil erosion and transport of nutrients to the bay prompted the Chesapeake Bay Commission to explore alternative scenarios for bioenergy cropping in the Chesapeake Bay watershed [82]:

1. 121, 500 additional ha of corn production under typical management
2. 121, 500 additional ha of soybean production under typical management
3. 121, 500 ha of switchgrass (converted from pasture and hayland) planted for perennial biomass energy crop with no nitrogen fertilizer
4. 121,500 ha of additional corn added but produced with best management practices such as cover crops and other technologies that reduce erosion and nutrient losses.
5. 405,000 ha of switchgrass (converted from pasture and hayland) planted for perennial biomass energy crop with no nitrogen fertilizer

**Figure 2.**
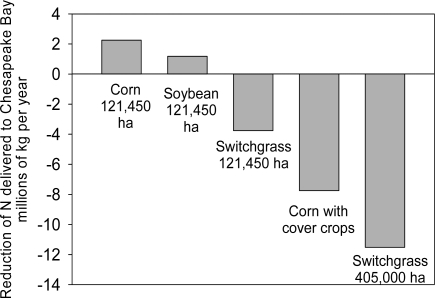
Estimated change in nitrogen loading to the Chesapeake Bay resulting from five cropping system scenarios: (1) 121, 500 additional ha of corn production under typical management; (2) 121, 500 additional ha of soybean production under typical management; (3) 121, 500 ha of switchgrass (converted from pasture and hayland) planted for perennial biomass energy crop with no nitrogen fertilizer; (4) 121,500 ha of additional corn added added but produced with best management practices such as cover crops and other technologies that reduce erosion and nutrient losses; (5) 405,000 ha of switchgrass (converted from pasture and hayland) planted for perennial biomass energy crop with no nitrogen fertilizer. Adapted from the Chesapeake Bay Commission [82].

The estimated changes in N load to the bay for each of the scenarios showed that simply adding more row crops with typical management increased N loading to the bay (Figure 2). However, replacing pasture and hayland with switchgrass grown without nitrogen fertilizer substantially reduced N loading as did adopting new BMPs and cropping practices for corn. Switchgrass, however, requires some nitrogen fertilizer input for production [50]. Therefore, growing switchgrass for biomass production with no nitrogen fertilizer input would be impractical.

The increased use of biomass energy crops has been recommended as a strategy for mitigating atmospheric increases in CO_2_ [83]. Advocates of biofuels consider them to have a near-zero net emission of GHGs because they recycle carbon, consuming it during crop production and releasing it as the fuels are used, as compared with fossil fuels, which release ancient carbon that was consumed by plants long ago. However, growing the crops requires energy (e.g., to operate farm machinery), as does converting the harvest into usable fuels. In the near term, CO_2_ can be sequestered in the soil during plant growth, thereby reducing GHG concentrations; in the long term, however, the soil's capacity to store carbon is limited. Along with soil carbon sequestration, coproducts of biofuel production, such as lignin and protein, can displace net GHG emissions, making these system sinks “carbon- or GHG-negative.” Coproducts can “remove CO_2_” from the atmosphere by displacing demand for fossil fuels, so as new technology is developed to extract more coproducts from biomass, this sink for CO_2_ will increase.

Increases in soil organic C under warm-season perennial grasses have been reported in several regions of the USA [84–87] and central Europe [88]. Potential benefits from C sequestration under perennial grasses depend on the cropping system used or replaced. In the southeastern USA, switchgrass grown for biomass accumulated more total soil C than cotton (*Gossypium hirsutum* L.) or corn row crops, but not as much as bahiagrass (*Paspalum notatum* Flugge) pasture [89]. Studies in Tennessee and Virginia, however, have shown that soil organic C concentrations and inventory under switchgrass were no different than beneath tall fescue (*Festuca arundinacea* Schreb.) or pasture [90]. Short-term (5- to 7-yr) soil C gains under switchgrass were minimal when it was managed for hay or grazing in Pennsylvania [91].

### 5.1 Greenhouse Gas Emissions from Perennial Bioenergy Crops

Agriculture is responsible for about 7% of total USA GHG emissions [92]. Proper management of agricultural systems can reduce direct emissions and offset emissions from other entities by sequestering C in the soil [93–95]. However, GHG emissions from USA forage and grazing lands have not been extensively quantified [96].

Biofuels have a large potential to reduce the GHG emissions associated with energy use [5]. Bioenergy cropping systems could help offset GHG emissions, but quantifying that offset is complex. Bioenergy crops offset CO_2_ emissions by converting atmospheric CO_2_ to organic C in crop biomass and soil, but they also emit N_2_O and vary in their effects on soil oxidation of methane. Quantifying these factors is necessary to determine the net effect of several bioenergy cropping systems on soil C sequestration and GHG emissions.

In bioenergy cropping systems, N_2_O emissions were the largest source of GHGs [5]. Nitrous oxide emissions result from N inputs from fixation, fertilization, above-ground residue, decomposition of below-ground residue, and mineralization of soil organic matter [97]. Management practices, such as tillage, also affect N_2_O emissions from the soil [98]. Fossil fuel inputs from agricultural machinery and chemical application are affected by both the choice of crop and management practices. Reducing farm operations through reducing tillage, planting, and N fertilizer use significantly reduced net GHG emissions [5, 94, 99, 100].

Accounting for the effects of global land use change in GHG emission calculations, however, significantly alters estimates of the global warming potential of bioenergy crops. Converting land currently in forest or grassland to bioenergy crop production incurs a large “carbon debt” that may take decades or centuries to repay [101]. Model simulations indicate that producing grassland biomass on marginal or degraded lands does not incur a carbon debt [101].

Other computer simulation analyses of land use change suggest that any diversion of cropland from food to bioenergy production in the USA will result in more land converted to food crop production in other countries. The associated land clearing and crop production will actually increase GHG emissions [68]. Thus, policy analysts recommend that emphasis be placed on using crop residues, municipal solid wastes, and other biomass sources that use carbon otherwise not incorporated into human food.

### 5.2 Energy Balance Metrics of Bioenergy Cropping

Life cycle assessment has been used to evaluate the environmental impacts of products through quantifying their energy and material flows at all stages [102]. The energy balance and a consideration of N cycles are some of the key elements in life cycle assessments of biofuels. The energy balance for switchgrass production considers the energy content of the biomass minus the fossil energy used in production (i.e., the net energy production from the system). Biomass can be directly combusted or the cellulose fraction can be converted to ethanol and the lignin fraction combusted. Producing ethanol from switchgrass results in an energy ratio (ratio of energy output vs. energy input; values greater than 1 imply energy output greater than input) of about 5.4 compared with 1.25 from corn grain [49]. This is because the corn stover is not included in the energy balance. Return of corn stover to the soil is needed to reduce soil erosion and maintain soil quality [103].

The N cycle has a significant impact on the energy balance and production of greenhouse gases. The fossil fuel energy required to produce N used in biofuel production can account for a significant portion of the total system energy requirements. Thus, reducing the amount of fertilizer N needed can significantly increase the energy balance (net production) of the system. Perennial forage crops can have lower requirements for N than an annual crop like corn, thereby reducing the fossil fuel energy requirement. Lower N use also reduces the emission of N_2_O, a potent greenhouse gas.

## 6. Implications of Large Scale Bioenergy Cropping with Perennial Forages

In the USA, national goals for renewable energy call for 20% of transportation fuels, 25% of chemicals, and 5% of the nation's power to come from biomass feedstock by 2030 [104]. Achieving these targets will require an annual biomass supply of 907 million Mg (1 billion tons) [60]. The biomass would come from crop residues, perennial energy crops, manures and other waste materials, and grains. The DOE-USDA group estimated that 22.3 million ha of the 181.4 million ha of USA cropland would be needed to produce perennial biomass feedstock. The land area was proposed to come from cropland pasture (9.1 million ha; permanent pastureland was excluded from the analysis), hayland (4.2 million ha; but not alfalfa hayland), CRP (4 million ha; only those acres suited to grass production), and reallocation of existing cropland (5.7 million ha).

**Figure 3.**
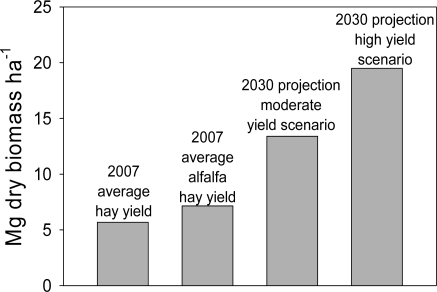
Comparison of 2007 average hay yields (Mg ha^−1^) in the USA with herbaceous biomass yields projected for perennial energy crops in 2030. Current hay yields are from the National Agricultural Statistics Service (www.nass.usda.gov) and projected 2030 biomass yields are from the “billion ton” report of Perlack *et al*. [60].

There were several assumptions made to meet those estimates. Key among them were (1) grain yields would increase by 50% by the year 2030; (2) technology will be developed to recover 75% of all annual crop residues; (3) all cropland would be managed via no-till; and (4) average annual biomass yields of 12.3 Mg ha^−1^ across all perennial bioenergy crop lands were achievable. Figure 3 illustrates the yield increases needed to reach assumption 4.

The billion-ton report also contained some very important caveats acknowledged by the authors, including the following (1) demand for meat production could increase, which would make conversion of cropland to perennial energy crops less likely; (2) higher export demand for some crops could limit cropland conversion to perennial crops; (3) the expected demand for forage would likely decrease because of the current trend of increasing the proportion of cattle in large confined animal feeding operations.

The assumptions and caveats have important implications for how existing forage and grazing lands are used in the future. For example, if the targets and technology are not met for grain yields and crop residue removal then much more land area will be required to produce the billion tons of biomass. Similarly, if the first two caveats reduce the likelihood of cropland conversion to perennial energy crops, then the production of perennial energy crops could be forced to more marginal lands. Similarly, the proposed replacement of 405,000 ha of pasture and hayland with switchgrass in the Chesapeake Bay region of the USA [82] would force forage-livestock production to other regions or cause greater intensification of confined animal production. Projections of expanded ethanol and biodiesel production in the USA to 60 billion gallons in 2030 indicate large reductions in pastureland acreage [105]. All of these aspects will place tremendous pressures on hay, forage, and pastureland in the future and the expanding land base necessary for biomass production would probably force forage and grazing lands production to ever more marginal lands. This could have very important implications for the forage-livestock industry. Shortages of forage crops and land for grazing livestock could result in higher production costs and reduce the profitability of livestock production. For example, De la torre Ugarte *et al*. [59] outlined such a scenario: with the introduction of bioenergy crops, traditional crops may lose acreage to the new crops, which would generate higher prices for the traditional crops and in turn there would be incentives to convert more idle land and pastureland to traditional crop production.

Progressing to the second generation of biofuels will require transitions in the forage-livestock industry and in agriculture as a whole to accommodate both fuel and food production. Perennial forages used as biomass feedstock crops are a key component of this transitional process. Farmers face several competing demands and pressures from markets, governmental policies, and society expectations in producing crops for food or fuel. It is unlikely that the quest for renewable fuels will end with the second generation. Developing and implementing renewable energy production systems based on sound environmental stewardship should be the legacy passed on by the second generation to future generations of renewable fuels.

## Figures and Tables

**Table 1.** Example biomass yields from selected perennial crops and lands.

			Biomass yield		
		N rate	Range	Mean	
Crop	Location and description	kg ha^−1^	----Mg ha^−1^----		Source
Switchgrass	Field-scale plots (3 to 9.5 ha) on 10 farms in Nebraska, South Dakota, and North Dakota USA harvested for 5 yr	0–212	5.2–11.1		49
*Miscanthus*	Experimental plots harvested for 3 yr in Denmark	60	1.4–18.2	9.1	106
*Miscanthus*	Experimental plots irrigated and harvested for 3 yr in Portugal	60	7.5–40.9	25.2	106
Reed canarygrass	Experimental plots in Indiana USA harvested for 3 yr	0–168	9.4–10.1	10.0	36
Reed canarygrass	Experimental plots at two sites in Iowa USA harvested for 5 yr	140	5.5–10.2	7.7	37
Alfalfa	Experimental plots at two sites in Minnesota USA harvested for 2 yr	0	7.0–12.0		42
Bermudagrass	Three experimental plot sites in Georgia USA for 3 yr	NR^1^	12.8–19.9	15.0	45
Napiergrass	Experimental plots in northern Florida USA harvested for 2 yr	200		46.3	107
Eastern gamagrass	Summary of studies from nine states in the eastern USA	84–301	6.5–15.9		47
Prairie cordgrass	Experimental plots in South Dakota USA harvested for 4 yr	0	4.6–8.6	6.4	48
Pasture on marginal land	10 pasture sites in southern Iowa USA	NR	0.8–8.2	4.2	58
CRP land	34 sites in seven northeastern USA states	0		6.6	61
CRP land	Experimental plots at three South Dakota USAsites harvested for 3 yr	0–224	2.5–6.0		62
Low-input high-diversity prairie	1 to 16 plant species grown in small plots grown for 10 yr at Cedar Creek, Minnesota USA	0		3.7	65

1 NR, not reported.

**Table 2.** Example biomass yields from several switchgrass cultivars in the USA, Canada, and Europe.

	North	South			Midwest	Pennsylvania		Mid-Atlantic states^7^			Alabama^9^		North			
Cultivar	Dakota^1^	Dakota^2^	Wisconsin^2^	Iowa^3^	States^4^	A^5^	B^6^	1-cut	2-cut	Texas^8^	A	B	Carolina^10^	Quebec^11^	Greece^12^	Italy^12^
	----------------------------------------------------------------------------------------------Mg dry biomass ha^−1^------------------------------------------------------------------------------------															
Cave-in-Rock	4.9	3.8	14.3	9.3	9.2	8.6	8.2	10.8	15.4	2.6			12.4	12.2	12.5	7.7
Dacotah	5.4	2.9	7.4													
Forestburg		3.9	9.4	6.9												
Shawnee	5.6	5.1	11.4	8.8		8.5										
Sunburst	7.4	4.6	11.5	6.8	8.8									10.6		
Trailblazer	6.9	4.6	11.0	7.9		6.7	12.4									
Alamo				12.1				15.2	16.3	15.3	23.0	12.9	14.2			
Pathfinder					8.3		11.0							11.5		
Shelter								10.3	13.6							
Kanlow							12.1	15.0	16.4	11.0	18.2	11.6			17.1	10.0
Blackwell					9.1											
NJ50							12.6									
Summer	5.5														14.6	7.4
BoMaster													15.8			
Performer													12.8			

1 [108];

2 [54];

3 [109];

4 [110];

5 [55];

6 Sanderson unpublished;

7 [111];

8 [21];

9 [19];

10 [23, 24],

11 [112],

12 [113].
